# Impact of Housing Condition on Welfare and Behavior of Immunocastrated Fattening Pigs (*Sus scrofa domestica*)

**DOI:** 10.3390/ani11030618

**Published:** 2021-02-26

**Authors:** Linda Steybe, Kevin Kress, Sonja Schmucker, Volker Stefanski

**Affiliations:** 1 Department of Behavioral Physiology of Livestock, Institute of Animal Science, University of Hohenheim, Garbenstraße 17, 70599 Stuttgart, Germany or kress@german-genetic.de (K.K.); sonja.schmucker@uni-hohenheim.de (S.S.); volker.stefanski@uni-hohenheim.de (V.S.); 2 German Genetic, Schweinezuchtverband Baden-Wuerttemberg e.V., Im Wolfer 10, 70599 Stuttgart, Germany

**Keywords:** immunocastration, vaccination, Improvac^®^, animal welfare, behavior, housing conditions, environmental enrichment, surgical castration

## Abstract

**Simple Summary:**

This study aimed to analyze if the well-known positive effects of immunocastration on the behavior and welfare of pigs are robust to varying environments. One hundred forty-four male pigs were studied with regard to their sex category (EM: entire males, IC: immunocastrates, BA: barrows) and housing environment (ENR: enriched, STD: standard, MIX: repeated social mixing). The second (=effective) vaccination was administered to the immunocastrates 5 or 6 weeks before slaughter according to the standard protocol. Regardless of housing conditions, beneficial effects of immunocastration on a reduction in undesired agonistic and sexual behavior, including penis biting and penile injuries, were observed. Enriched housing showed a beneficial effect on play, whereas the social mixing environment reduced the number of social nosing events. The novel result is that the beneficial effects of immunocastration on behavioral and welfare aspects apply independent from the housing environments assessed in this study.

**Abstract:**

The aim of this study was to investigate whether the well-known positive effects of immunocastration on the behavior and welfare of pigs persist under varying environments. One hundred forty-four male pigs were studied with regard to their sex category (EM: entire males, IC: immunocastrates, BA: barrows) and housing environment (ENR: enriched, STD: standard, MIX: repeated social mixing). The vaccination of immunocastrates included two injections at the age of 12 and 22 weeks. Regardless of the housing conditions, frequencies of sexual and fighting behavior expressed by immunocastrates shifted from boar-like to barrow-like behavior after the second immunocastration vaccination (Mixed model analysis, *p* < 0.05). Penis biting decreased in IC after the second vaccination (Wilcoxon signed-rank tests, *p* = 0.036) and penile injuries were lower in IC animals compared to EM (Mixed model analysis, *p* < 0.001). Housing-dependent effects on behavior could also be observed in the animals at a relatively young age. Enriched housing showed a beneficial effect on play behavior (Chi-square test, *p* < 0.001) and the social mixing environment reduced the number of social nosing events (Mixed model analysis, *p* < 0.05). The positive effects of immunocastration thus are robust to all housing conditions assessed in this study.

## 1. Introduction

Male piglets reared for pork production have been surgically castrated for centuries worldwide, and this surgical castration is in most cases carried out without anesthesia or analgesia. Even though castration is known to be stressful and painful for the animal [1,2,3] and despite the commitment of European stakeholders of the pork production chain to ban this practice from 2018 onwards, about 60% of male piglets are still surgically castrated in Europe [4] to avoid boar-taint, an unpleasant off-odor which can occur after the onset of puberty and significantly reduces pork eating quality [5]. Castration is also performed in order to inhibit the display of boar-specific agonistic and sexual behavior [6,7]. Aggressive behavior of entire males includes biting of the penis [8,9] and other parts of the body of conspecifics. The resulting skin lesions [10] are potential entry points for pathogens and thus pose a potential health risk [11]. Losing fights and an accumulation of wounds in general can have an overall negative effect on the affective state of an animal, thus resulting in welfare concerns [12]. Penile injuries lead to inflammations making penis biting a severe welfare problem as well [9]. In addition, the experience of being mounted by another pig is uncomfortable for the recipient and often accompanied by screaming [13], an indicator of the animal’s welfare being compromised [14].

Castration was for a long time the management tool of choice to address those problems, but with immunocastration (IC), an alternative to surgical castration is now available. The principle of IC is an active immunization against the body’s endogenous gonadotropin-releasing hormone (GnRH), which controls testicular functions and testosterone release. The vaccination with Improvac^®^ (Zoetis Inc., Louvain-la-Neuve, Belgium) suppresses testicular functions for several weeks after the second vaccination [15], and causes a reduction in aggressive and sexual behavior [7]. Kress et al. [16] showed in a companion project that physiological changes associated with immunocastration are not impacted by housing condition. Von Borell et al. [17] have summarized welfare aspects of entire male pigs and immunocastrates, and several studies have comprehensively analyzed the behavior of boars, immunocastrates, and barrows [6,7,15,18,19]. Knowledge about the modulating effects of the housing environment on behavior and welfare of immunocastrates in comparison to surgical castrates (barrows) and boars, however, is still limited.

Housing conditions are generally known to considerably influence the behavior of animals. Environmental enrichment is often used to improve welfare, as it allows the animal to perform its natural behavior [20,21]. The multidimensional welfare concept by David Fraser [22] includes natural living as an integral part. Already in 1981, explorative behavior was recognized as a behavioral need of pigs [23]. Pigs will perform it regardless of the housing environment, which in barren environments can result in increasingly redirecting explorative behavior towards pen mates [24,25]. In turn, environmental enrichment has the potential to shift aversive pen-directed behavior towards the enrichment [20,26]. Pigs housed in enriched environments are furthermore found to be less fearful than pigs housed in barren pens [27], which can exhibit symptoms of chronic stress [28], are less active [20,29] and show less play behavior [30]. Play mainly occurs in the absence of immediate threats or pain and suffering, and could therefore represent an indicator of good welfare [31,32].

The aim of this study was to analyze the influence of various housing conditions on behavior and welfare aspects in male fattening pigs from different sex categories—surgical castrates (barrows; BA), entire males (EM), and immunocastrates (IC). When focusing on IC, the specific question was if the well-known positive effects of immunocastration on behavior and welfare persist under varying environments. In all three sex categories, we expected beneficial effects in enriched, but negative effects under stressful (social mixing) housing conditions on behavior and welfare in comparison to conventional (standard) housing. We further expected that entire males (EM) would benefit the most from the enriched conditions, as undesired aggressive and sexual behavior occurs particularly in this sex category. In an integral approach, behaviors from different behavioral groups and data on the individual animal level were used and validated with physiological parameters.

## 2. Materials and Methods

As part of the SusAn ERA-NET project “Sustainability in Pork Production with Immunocastration (SuSI)”, the behavior of male pigs was observed within a larger physiological study [16]. The main study goal was to test the reliability of immunocastration under various housing conditions.

### 2.1. Animals and Experimental Setup

The experimental protocol for our experiment (ID HOH47/17TH) was approved by the ethical committee for animal experiments of the regional authority of Tuebingen, Germany, and all procedures were conducted in accordance with the German Animal Welfare Act. A total of 144 male pigs of a standard German commercial genetic type (Piétrain × German Landrace) were studied in two consecutive trials (trial 1: November 2017–February 2018; trial 2: April 2018–August 2018). Piglets were born at the experimental unit of the University of Hohenheim (Unterer Lindenhof, Eningen, Germany) and housed under identical conventional farrowing conditions followed by flatdeck housing until the beginning of the experimental phase. The experiment started when the pigs were 10 weeks old and was continued until slaughter at an age of 27 or 28 weeks. Three days after birth, piglets were randomly allocated to 9 different experimental groups according to the Latin Squares method. They were assigned to three different sex categories (entire males/boars (EM): n = 48, immunocastrates (IC): n = 48, and surgical castrates/barrows (BA): n = 48), which will be also referred to as “sex”. Barrows were surgically castrated within the first week of life without anesthesia but received 0.2 mL Metacam^®^ (Meloxicam, 0.5 mg/mL, Boehringer Ingelheim Vetmedica, Ingelheim/Rhein, Germany). Immunocastrates received the first Improvac^®^ vaccination in the 12th (V1) and the second Improvac^®^ vaccination in the 22nd (V2) week of life. According to this experimental design, animals or pens of the sex category IC are either designated as IC_(V1)_, referring to the period before V2 or IC_(V2)_, referring to the period between 7 days after V2 until slaughter. Entire males remained untreated.

**Figure 1:**
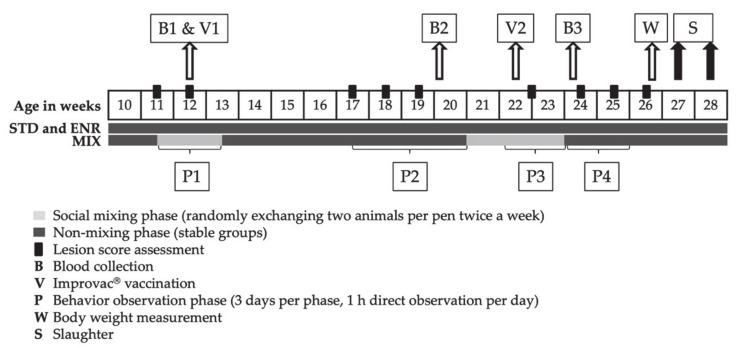
Experimental design of the study. Displayed are the animal’s weeks of age. Weeks, in which behavior observations occurred, are indicated with curly brackets. Mixing phases, where animals were exchanged in MIX pens every third day, are highlighted under the timeline in light grey, weeks without mixing have a dark grey background. Phases P1–P4 represent the following time points: P1: Pigs are about 80 days old, mixing carried out in the MIX pens, P2: Pigs are about 130 days old, no mixing carried out in the MIX pens, P3: Pigs are about 160 days old, mixing carried out in the MIX pens, P4: Pigs are about 170 days old, no mixing carried out in the MIX pens.

The three sex categories were subdivided and further allocated to three different housing environments, which will be further referred to as enriched (ENR, n = 36), standard (STD, n = 36), and mixing (MIX, n = 72). Each pen was equipped with a solid floor, a nipple drinker, minimal litter and a metal chain with an organic element as manipulable material. All pigs were provided ad libitum access to feed with the same standard diet [33] and provided with natural light. Differences with regard to housing conditions resulted from different space allowances in the pens (STD and MIX: 1.2 m^2^/pig indoors, ENR: 2.8 m^2^/pig indoors plus 3.2 m^2^/pig outdoor area) and from trough length and thus the possibility of synchronous feeding of all 6 animals in the pen (STD and MIX: not given with 20 cm/pig, ENR: given with 40 cm/pig). Social mixing (MIX) consisted of interchanging two pen mates within each sex every third day and was carried out within two weeks around the first Improvac^®^ vaccination (five mixing events) and three weeks around the second vaccination (eight mixing events) of immunocastrates, whereas EM-MIX and BA-MIX pens were mixed at corresponding time points. For this reason, two MIX pens existed per sex category and trial, resulting in double the number of animals for the MIX treatment. Animals were interchanged between those two pens throughout the respective trial. No littermates were present within any given pen of pigs. Animals were housed in groups of six. The health status of the animals was assessed twice a day by trained personnel. On the experimental unit a comprehensive hygiene framework was applied, including working after the all-in/all-out principle to cut off infection cycles and working in closed on-farm cycles by restocking breeding animals from the own herd. The piglets get vaccinated against Mycoplasma hyopneumoniae at the age of 3 weeks. The experimental design of the study is given in Figure 1.

### 2.2. Blood Collection (B1–B3)

In total, three blood samples were taken from each pig throughout the fattening period (Figure 1). Animals were separated individually and fixated by a snare pole. Blood was collected via puncture of the external jugular vein (Vena jugularis externa) into heparinized vials. The samples were centrifuged, and plasma was removed immediately from the blood cell interface. Samples were subsequently stored at –20 °C until further analysis.

### 2.3. Behavioral Observations (P1–P4)

To ensure individual recognition, each pig was tagged with stock marker spray on its flanks (Animal Marking Spray, Raidex, Dettingen/Erms, Germany). Behavior sampling was conducted by visually monitoring the behavior of pigs by a single trained observer. To ensure consistent coding of behavior throughout each trial, the same video (15 min) showing pig behavior of a pen in the first trial was watched four times (mean kappa = 0.955). Behavioral observations were carried out in four observational phases (P1–P4) throughout the fattening period (Figure 1). Each observation phase consisted of 3 observation days. Each pen was observed for one hour per day, split into periods of 30 min in the morning (between 8.00 h and 12.00 h) and 30 min in the afternoon (between 12.00 h and 16.00 h) in random order. To be able to observe all pens on one day by one observer, two pens were observed at the same time. With a total number of 12 pens per trial, this resulted in 3 h of observation in real time in the morning and in the afternoon. Synchronously to the indoor observations, the outdoor areas of ENR pens were monitored by video camera and analyzed later, and the respective information added to the dataset. Applying the behavior sampling method, occurrences of behaviors were recorded via direct observation together with details about which individuals were respectively involved [34]. By definition, a new behavior was scored after 3 s of pausing a previous behavior. The behavior groups of scored behaviors are listed below. The full ethogram is given in Supplementary Material (Table S1).

#### 2.3.1. Agonistic Behavior

- Total aggressive behavior includes all aggressive behavior elements recorded: reciprocal pressing (inverse or parallel), reciprocal pressing-cum-bite (inverse or parallel), head knock, head-knock-cum-bite, levering, chasing, biting (attempt), penis biting (attempt);
- Highly aggressive behavior: pressing-cum-bite, head-knock-cum-bite, biting (attempt), penis biting (attempt);
- Fighting: reciprocal pressing and pressing-cum-bite in parallel (heads beside heads) or inverse parallel (heads to tails) orientation;
- Total defensive behavior includes all defensive behavior elements recorded: retreat, fleeing, and mounting escape (attempt);
- Mounting escape (attempt): (trying to) retreat from being mounted.

#### 2.3.2. Sexual Behavior

- Total sexual behavior includes all sexual behavior elements recorded: anal–genital nosing, mounting (attempt), mounting with pelvic thrusts, mounting with an extended penis, mounting with pelvic thrusts, and extended penis;
- Mounting behavior: all types of mounting behavior, excluding mounting attempts;
- Highly sexual behavior: mounting with pelvic thrusts, mounting with an extended penis, mounting with pelvic thrusts and extended penis.

#### 2.3.3. Other Behaviors

- Abnormal stereotypic behavior: body-nosing, chewing ear, chewing tail, chewing pen mate (without tail and ear);
- Social nosing: nose approaches the body of another pig slowly within 5 cm, excluding the anal–genital region, no agonistic behaviors follow for at least 3 s;
- Play behavior: scamper play, pivot, head toss, flop, paw.

### 2.4. Skin Lesions

The number of integument lesions was assessed at 9 time points for each pig (Figure 1). The measurement was implemented as described in Table 1 on the basis of Schrader et al. [35]. Each side of the torso and each ear, as well as the tail, were separately assessed. In the results, lesion scores are displayed for the left body side only, as there were no differences between values for the left and right side (*t*-test, *p* > 0.05).

### 2.5. Penile Injuries

After slaughter, a physical examination of penile injuries of entire males and immunocastrates (n = 96) was performed as described by Weiler et al. [9]. For this purpose, the penis was dissected by gently pushing it towards the caudal direction within the preputium with two fingers to dissect the preputial sheet without causing fresh wounds to the penis. The penis was taken out of the prepuce and the numbers of injuries on the glans penis were counted. Fresh red wounds, severe gaping wounds and scars were counted and summed up to tally the total number of penile injuries. Wounds were identified by visual inspection and scars as bulging tissue were detected by palpating the glans penis with thumb and index finger.

### 2.6. Plasma Cortisol and Plasma Testosterone Level

Cortisol and testosterone concentrations in the plasma of the animals were determined using radioimmunoassay (RIA). The procedures themselves, as well as intra- and inter-assay variances, are described in detail in the study by Kress et al. [16], which drew on the same animals that were used in our study.

### 2.7. Body Weight

Individual body weight was measured when pigs were 180 days old, about 1 week before slaughter.

### 2.8. Data Processing and Statistical Analysis

Data processing of behavioral data, except for play behavior, penis biting and penile injuries, included the calculation of mean frequencies of behaviors per hour for each animal and observation phase. The experimental unit was defined as pig within treatments sex and housing. In the raw data, numbers of behavior occurrences were available for every animal during one hour of observation on each observation day. For each animal, frequencies of behaviors were averaged among the three observation days within one phase. Due to the high presence of non-occurrences (value = zero), play behavior was analyzed as a binomial variable. Per definition, animals either showed play on an observation day (1), or not (0). The response variable was the frequency of animals per treatment combination sex and housing. Calculated was the percentage of animals per treatment showing play on one observation day. Penis biting events prior to the second vaccination (P1 + P2) were summed up for each animal within treatments sex and housing and were compared to the sum of post vaccination (P3 + P4) observations. Events were summed up to increase the frequency of the behavior. Penile injuries were evaluated once per animal after slaughter. 

Statistical analysis was conducted using SAS Version 9.4 (SAS Institute Inc., Cary, NC, USA). Behavioral data of frequencies per observation phase and the sum of penile injuries after slaughter were analyzed as dependent variables with a mixed linear model (GLIMMIX procedure).

Fixed effects included sex category and housing environment as well as their interaction. The following model was used:

Y_ijklmn_ = μ + α_i_ + β_j_ + (αβ)_ij_ + γ_k_ + δ_l_ + (γδ)_kl_ + ϕ_m_ + (χϕ)_nm_ + (λγδ)_okl_ + ε_ijklmno_ (1)

with Y_ijklmn_ = response variable; μ = overall mean; α_i_ = effect of sex (fixed); β_j_ = effect of housing environment (fixed); (αβ)_ij_ = interaction of sex and housing environment (fixed); γ_k_ = effect of trial (random); δ_l_ = effect of pen (random); (γδ)_kl_ = interaction of trial and pen (random); ϕ_m_ = effect of sire (random); (χϕ)_nm_ = nested effect of dam within sire (random); (λγδ)_okl_ = nested effect of animal within pen and trial (random); ε_ijklmno_ = residual error.

Degrees of freedom were determined using the Kenward-Roger method and variance components were estimated using the restricted maximum likelihood (REML) method. Behavioral data were log-transformed after graphical inspection of residual plots. Post-hoc calculation of pairwise comparisons was conducted using the LSMEANS procedure and Bonferroni correction to adjust for multiple comparisons. Relative frequencies of animals per treatment combination showing play behavior were analyzed using the Chi-Square test (GLIMMIX procedure) and Tukey correction on treatment combination level. Fixed effects included trial, sex category, housing environment as well as the interaction of sex category and housing environment and a random overdispersion parameter. Lesion scores were analyzed per score in the same way as play behavior per treatment combination. Penis biting was analyzed within sex category (for dependent samples) and within observation phases using Wilcoxon signed-rank tests.

The relationship between each physiological parameter and behavioral parameters were analyzed calculating linear regression equations for every treatment combination using the method of analysis of covariance and the MIXED procedure. Testosterone and cortisol concentrations of blood sample B3 and the body weight (Figure 1) were used and inserted as a covariate into the model (1) respectively. Total aggressive, fighting, total sexual, mounting, abnormal and total defensive and mounting escape behaviors from observation phase P4 were inserted as dependent variable.

Significant differences are indicated by asterisks (*** *p* < 0.001, ** *p* < 0.01, * *p* < 0.05) or *p*-values given.

## 3. Results

### 3.1. Behavior

**Figure 2:**
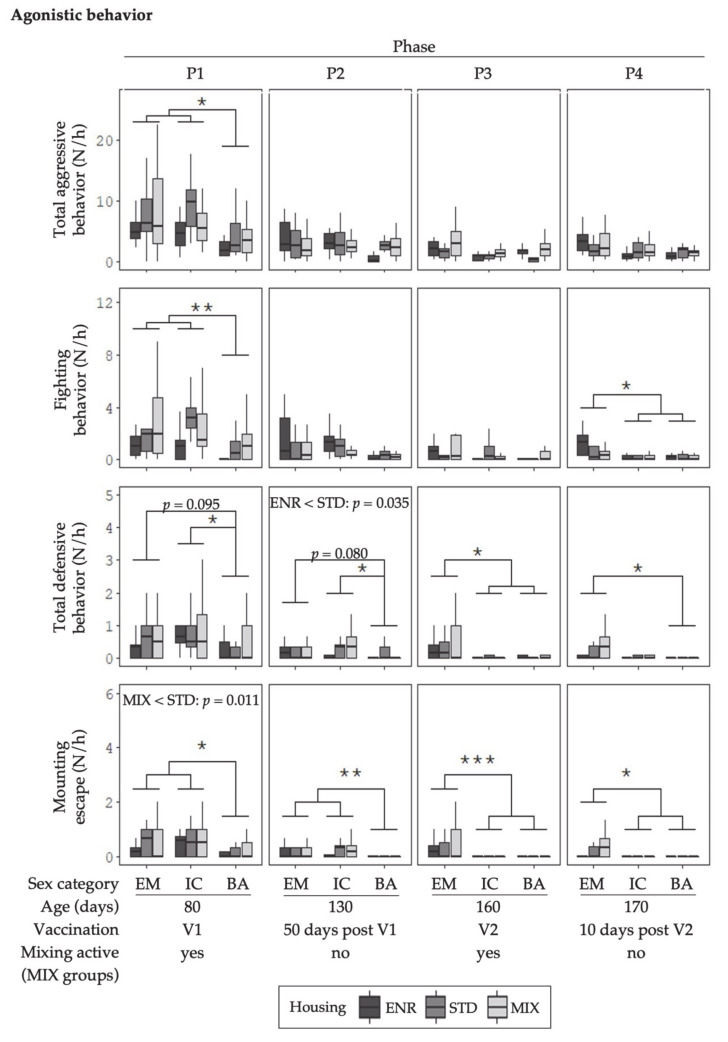
Mean number of occurrences (N) of agonistic behaviors per hour (h) for each animal are given as boxplots for the sex categories (EM = entire males, IC = immunocastrates, BA = barrows) in the different environments (ENR = enriched, STD = standard, MIX = mixing). Columns represent the data of animals in four behavior observation phases (P1–P4). V1 represents phases before, and V2 after the second immunization of IC. The MIX pens were mixed in P1 and P3. Note: no mixing was done in STD and ENR at any time. Significant differences between sex categories are indicated by asterisks (*** *p* < 0.001, ** *p* < 0.01, * *p* < 0.05). Housing differences are annotated with *p*-values in the graphs respectively. Boxplots show the minimum value with respect to the interquartile range, the 25th percentile, the median, the 75th percentile and the maximum value with respect to the interquartile range.

**Figure 3:**
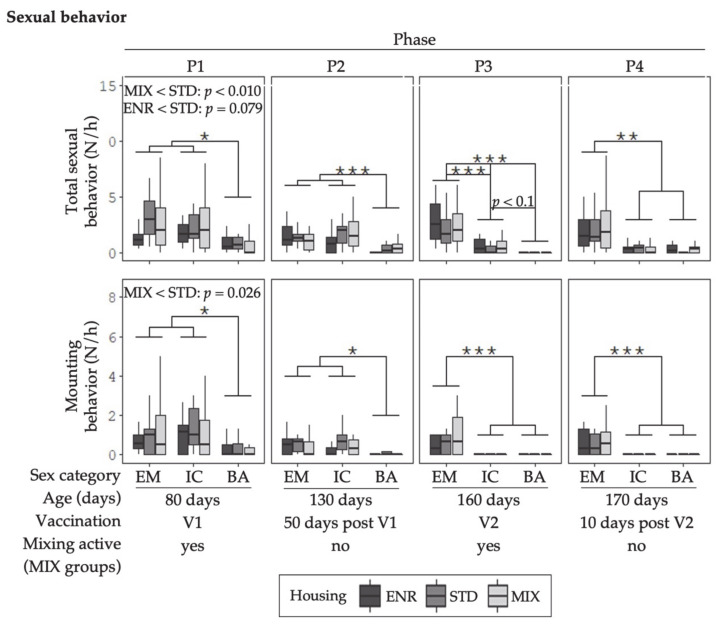
Mean number of occurrences (N) of sexual behaviors per hour (h) for each animal are given as boxplots for the sex categories in the different environments. Columns represent the data of animals in four different behavior observation phases (P1–P4). V1 represents phases before, and V2 after the second immunization of IC. The MIX pens were mixed in P1 and P3. Note: no mixing was done in STD and ENR at any time. Sex categories were: EM = entire males, IC = immunocastrates, BA = barrows; Housing environments were: ENR = enriched, STD = standard, MIX = mixing. Significant differences between sex categories are indicated by asterisks (*** *p* < 0.001, ** *p* < 0.01, * *p* < 0.05). Housing differences are annotated with *p*-values in the graphs respectively. Boxplots show the minimum value with respect to the interquartile range, the 25th percentile, the median, the 75th percentile and the maximum value with respect to the interquartile range.

**Figure 4:**
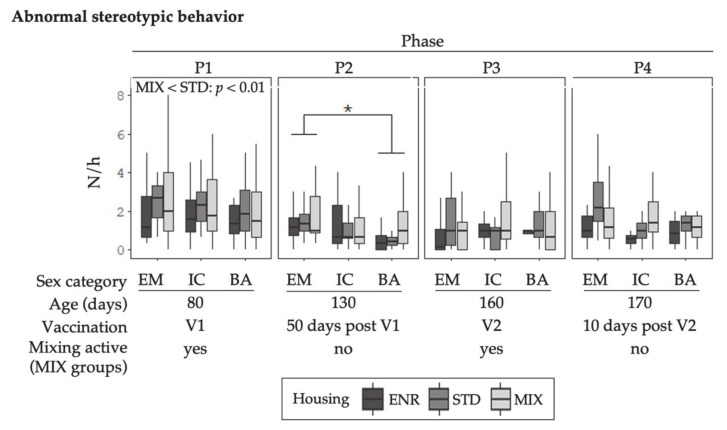
Mean number of occurrences (N) of abnormal stereotypic behaviors per hour (h) for each animal are given as boxplots for the sex categories in the different environments. Columns represent the data of animals in four different behavior observation phases (P1–P4). V1 represents phases before, and V2 after the second immunization of IC. The MIX pens were mixed in P1 and P3. Note: no mixing was done in STD and ENR at any time. Sex categories were: EM = entire males, IC = immunocastrates, BA = barrows; Housing environments were: ENR = enriched, STD = standard, MIX = mixing. Significant differences between sex categories are indicated by asterisks (*** *p* < 0.001, ** *p* < 0.01, * *p* < 0.05), whereas for tendencies *p*-values are given. Housing differences are annotated with *p*-values in the graphs respectively. Boxplots show the minimum with respect to the interquartile range, the 25th percentile, the median, the 75th percentile and the maximum value with respect to the interquartile range.

**Figure 5:**
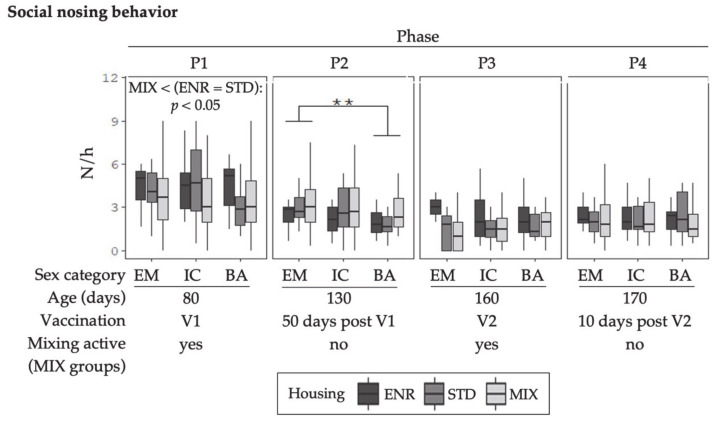
Mean number of occurrences (N) of social nosing per hour (h) for each animal are given as boxplots for the sex categories in the different environments. Columns represent the data of animals in four different behavior observation phases (P1–P4). V1 represents phases before, and V2 after the second immunization of IC. The MIX pens were mixed in P1 and P3. Note: no mixing was done in STD and ENR at any time. Sex categories were: EM = entire males, IC = immunocastrates, BA = barrows; Housing environments were: ENR = enriched, STD = standard, MIX = mixing. Significant differences between sex categories are indicated by asterisks (*** *p* < 0.001, ** *p* < 0.01, * *p* < 0.05), whereas for tendencies *p*-values are given. Housing differences are annotated with *p*-values in the graphs respectively. Boxplots show the minimum value with respect to the interquartile range, the 25th percentile, the median, the 75th percentile and the maximum value with respect to the interquartile range.

The behavioral data illustrated in Figure 2, Figure 3, Figure 4 and Figure 5 represent mean frequencies of behaviors per hour for each animal. The graphs display the different sex and housing treatment combinations in the four behavior observation phases (P1–P4). As there was no significant sex x housing interaction, the effects for sex and housing are reported separately.

#### 3.1.1. Agonistic Behavior

An effect of sex was found for several agonistic behaviors (Figure 2). In the period before the second vaccination of immunocastrates (V1; P1 and P2), mounting escapes were more often displayed by EM and IC_(V1)_ than by BA, total aggressive behavior and fighting were greater in P1. Tendencies (*p* < 0.1) for greater numbers of total defensive behavior by EM compared to BA were observed in P1 and in P2. After the second vaccination (V2; P3 and P4) the pattern changed. Now, IC_(V2)_ and BA displayed lower levels of total defensive behavior (P3) and fighting (P4) as well as mounting escapes (P3 and P4) than EM.

Housing influenced agonistic behavior, whereby the number of mounting escapes were greater in STD when compared with MIX (P1) and the number of total defensive behaviors was greater in STD when compared with ENR housing type (P2).

There were no differences regarding sex or housing for highly aggressive behavior (Supplementary Material, Figure S1).

#### 3.1.2. Sexual Behavior

Before the second vaccination (V1; P1 and P2), EM and IC_(V1)_ showed greater numbers of total sexual behavior and mounting than BA (Figure 3). The pattern of total sexual behavior changed in IC with the second vaccination (V2). In P3, EM showed the greatest and BA the lowest numbers. The sex category IC_(V2)_ displayed less numbers than EM and there was also a tendency for IC_(V2)_ to show more total sexual behavior than BA. In P4, both IC_(V2)_ and BA showed low numbers of total sexual behavior and mounting and differed significantly from EM. A similar effect was observed for highly sexual behavior in P3 (Supplementary Material, Figure S2).

Housing conditions had an effect on total sexual and mounting behavior in P1. Total sexual behavior was shown more often in STD animals than in MIX animals. A similar tendency (*p* = 0.079) was observed between STD and ENR animals. Mounting was displayed more frequently in STD housing as compared to MIX housing.

#### 3.1.3. Abnormal Stereotypic Behavior

An effect of sex was observed for abnormal stereotypic behavior in P2 (Figure 4). The sex category EM showed greater numbers than BA.

In P1, a housing effect was noted for abnormal stereotypic behavior in a way that STD animals showed it more often compared to MIX animals.

#### 3.1.4. Social Nosing

The sex category influenced social nosing in P2, whereby EM showed greater numbers than BA (Figure 5).

A housing effect was observed in P1 for social nosing and was shown to occur least frequently in MIX pens.

#### 3.1.5. Play Behavior

**Figure 6:**
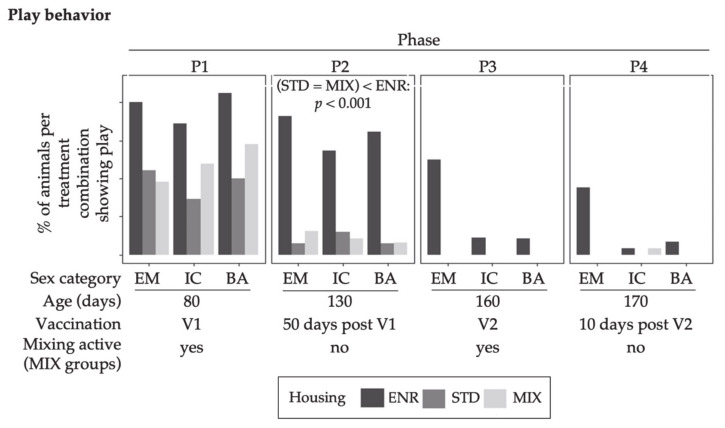
Percentages of animals per treatment combination that demonstrated play behavior at least once when assessed for an hour daily for three observation days. Columns represent the data of animals in four different behavior observation phases (P1–P4). V1 represents phases before, and V2 after the second immunization of IC. The MIX pens were mixed in P1 and P3. Note: no mixing was done in STD and ENR at any time. Sex categories were: EM = entire males, IC = immunocastrates, BA = barrows; Housing environments were: ENR = enriched, STD = standard, MIX = mixing. Housing differences are annotated with *p*-values in the graphs respectively.

Play behavior was infrequently observed and not displayed by all animals. Figure 6 represents the percentages of animals per treatment combination showing play at least once on an observation day. There was no specific effect of sex on the relative frequencies of animals showing play behavior.

However, an effect of housing existed in P2. The share of ENR animals showing play behavior was greater than for STD and MIX treatments.

### 3.2. Lesion Scores

**Figure 7:**
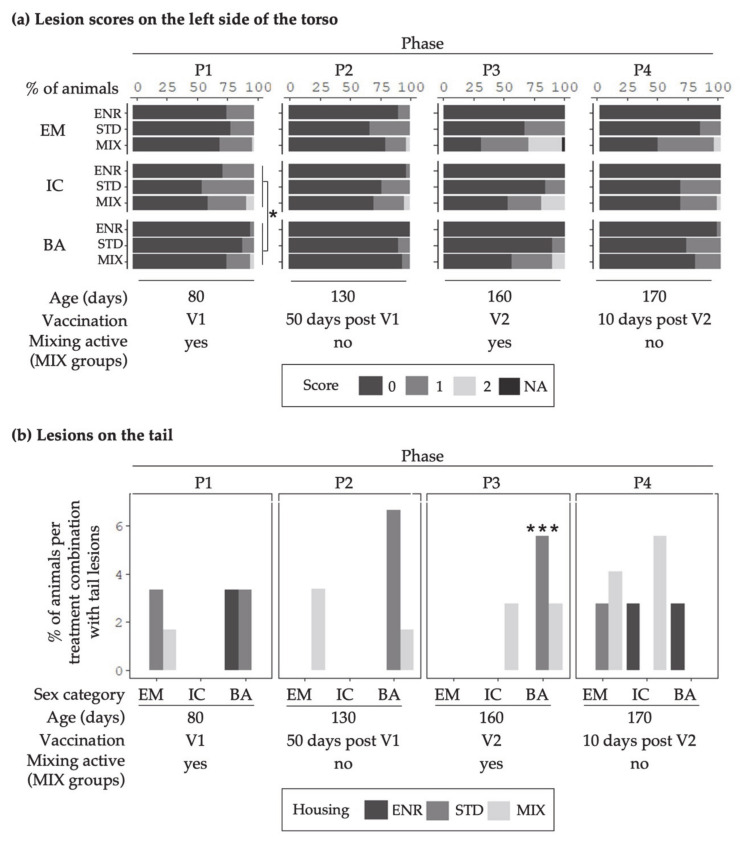
Percentages of animals of the treatment combinations (Sex categories: EM = entire males, IC = immunocastrates, BA = barrows; Housing environments: ENR = enriched, STD = standard, MIX = mixing) with (**a**) certain lesion scores on the left side of the torso and (**b**) lesions on the tail (score 1). Columns represent the data of animals in four different behavior observation phases (P1–P4). V1 represents phases before, and V2 after the second immunization of IC. The MIX pens were mixed in P1 and P3. Note: no mixing was done in STD and ENR at any time. Significant differences between treatment combinations are indicated by asterisks (*** *p* < 0.001, ** *p* < 0.01, * *p* < 0.05).

Figure 7 shows the percentages of animals per treatment combination with certain skin lesion scores on the left side of the torso (a) and on the tail (b). Skin lesions occurred regularly in all sex and housing treatments. Tail lesions (score 1) were seen only in a small percentage of animals.

A significant effect of sex was found for score 0 and 1 in P1 for the torso (*p* ≤ 0.02). IC_(V1)_ showed more injuries than BA.

In P3, an effect of the interaction sex x housing was found for the tail lesion score (*p* < 0.001). In the treatment combination BA from the STD environment, more tail lesions were observed compared to other treatment combinations (*p* < 0.001).

With regard to ear assessment, statistical analyses revealed no sex or housing effects.

The trial itself did have an effect on the numbers of skin lesions as fewer lesions were observed in the second trial (*p* < 0.05).

### 3.3. Penis Biting and Penile Injuries

Numbers of penis biting incidents per animal are shown in Table 2. After the second vaccination (postV2), EM animals did show more penis biting than IC (*p* = 0.039). Numbers did decrease for IC from pre to post V2 (*p* = 0.036) but did stay at the same level for EM throughout.

Mixed model analysis revealed a significant effect of sex (*p* < 0.001) on the occurrence of penile injuries per animal, which were examined after slaughter (Table 3). Post-hoc testing revealed that EM showed double the number of injuries compared to IC. The treatment combination EM-MIX appeared to have greater numbers of injuries compared to other EM and all IC treatment combinations, but the level of significance was not reached for an effect of housing (*p* = 0.100).

### 3.4. Linear Regression Analysis—Body Mass and Physiological Parameters

The overall effects of sex and housing conditions on growth performance, cortisol and testosterone concentrations in the animals are described in the companion study by Kress et al. [16]. The growth performance of IC_(V2)_ was significantly higher than of EM and BA. In the finishing phase, growth performance of MIX pens was lower compared to ENR pens, while STD animals were in-between. Live weight of the animals at the slaughterhouse, assessed directly before bleeding, was 124.7 ± 10.3 kg (mean ± standard deviation, n = 108 animals).

The present study, on the other hand, investigates in more detail the degree to which body mass, cortisol and testosterone relate to the number of specific behaviors displayed by the individual animals at the end of the experiment. Linear regression analysis revealed three significant relationships between individual behavior and physiological parameters assessed in this study. Significant regression equations were found for the relationship between the number of mounting escapes and body weight (F_(9,118)_ = 2.47, *p* = 0.013, R^2^ = 0.3155). All regression equations with the dependent variable body weight are to be found in Supplementary Material, Table S2. The animal’s cortisol concentration in plasma related significantly to the number of total aggressive (F_(3,116)_ = 2.89, *p* = 0.038, R^2^ = 0.0231) and fighting behavior (F_(3.123)_ = 5.20, *p* = 0.002, R^2^ = 0.2908). Regression equation information are given in Supplementary Material, Table S3. No significant relationships were found between the behaviors and testosterone concentration in plasma (Supplementary Material, Table S4).

## 4. Discussion

It is quite clear today that immunocastration has many positive effects on behavioral and welfare aspects in male pigs [6,7,15,19]. New results of the present study are that these beneficial behavioral effects of immunocastration apply independent of the housing environments, and these findings are relevant for various farming systems, where pigs are reared segregated by sex. They may be of high relevance for conventional farm conditions, but also for farms working with enriched conditions with varying climatic situations due to outdoor access, such as organic farming. The reliability of the behavioral effect underlines the usefulness of IC for different housing systems.

In general, the present study confirms a well-known behavior shift from boar-like to a barrow-like behavior in IC after the second vaccination, which is characterized by a reduction in aggressive and sexual behavior [6,7,15,19,36,37]. Of particular relevance is that the frequency of the highly problematic behavior penis biting declined after the second vaccination in IC animals, whereas this aversive behavior did not change in EM over time. Confirming previous observations [8,9], resulting penile injuries were consequently more frequent in EM than in IC. This finding addresses the knowledge gap described by von Borell et al. [17] who called for an assessment of the modulating effects of housing conditions on penile injuries in EM. In our study, we did not see a reduction in the number of penile injuries observed in EM animals due to enrichment.

The main focus of the present study, however, was to investigate whether the beneficial effects of immunocastration seen under standard housing conditions are also evident in enriched and stressful environments. Potentially stressful mixings in this study did not interfere with the positive effect of IC with respect to the prevention of penile injuries and other undesired behaviors such as mounting. This is an important aspect, as management procedures under practical farm condition also include changes in group composition. It must be emphasized, however, that the magnitude of physiological and morphological alterations in the animals in the present report, like low share of lesion score 2 (more than 15 scratches) even in MIX pens, point to mild and/or moderate stress conditions. Thus, we cannot conclude that stressors, per se, have no effect on immunocastration. Another important conclusion from the present findings is that even for IC kept in enriched housing with outdoor access, such as under organic farming, the potential microbial challenges posed by outdoor areas/access [38] did not have any impact on the effectiveness of immunocastration as such.

The companion study by Kress et al. [16], using the same animals, quantified immune responses of IC by measuring GnRH-binding and analyzed its consequences for testosterone concentrations. The study showed that from a physiological point of view, immunocastration is a reliable procedure and works under different housing conditions. Other studies with the same set of animals showed that the downregulation of testicular functions and androsterone worked well with regards to carcass and meat quality traits [39,40].

Together, above mentioned studies [16,39,40] and the present study allow an overall assessment of IC under varying housing conditions. The beneficial effects of immunocastration on behavior, physiology, morphology and animal welfare are independent of the housing environments. In our study we assessed the animals in groups of six per pen, whereas many farms work with larger group sizes, which has to be noted. The method of behavior sampling allows for scoring behaviors that are infrequently displayed and/or of short duration, like in many social interactions. A potential disadvantage of scoring behaviors in larger groups of animals simultaneously is that subtle behaviors, e.g., social nosing, might been overlooked in some cases. Thus, it cannot be completely ruled out that the frequency of subtle nosing behavior was underestimated in the present report. We however see the clear advantage of behavior sampling with recording of all occurrences as this assures that important overt social behavioral elements are not missed. The agonistic, sexual or play behavior recorded here is characterized by distinct body movements and was explicitly identifiable in the groups of animals.

The housing conditions investigated in the present study revealed some differences in behavioral profiles, particularly in the first two observation phases. This likely reflects an effect of age. Noteworthy is the beneficial effect of enriched housing. Under enriched conditions, play behavior was displayed by more animals than under conventional or mixed conditions. Play is considered as an indicator of positive affective states [41,42] and consequently less often expressed in rather adverse environments [31,32]. In line with these observations are the lower incidents of defensive behavior in enriched housing. Rushen et al. describe defensive behavior as a tool to reduce the number of times of being bitten and to prevent further aggression [43]. The difference in defensive behavior in our study indicates that limited space under STD housing made active withdrawals more often necessary. However, a general effect of housing was not observed on total aggressive behavior and was found only in the first observation phase for total sexual behaviors, which partially supports Tallet et al. [29] with respect to aggressive behavior of EM under enriched conditions (a reducing effect of enrichment was, however, observed in BA). In contrast, van de Weerd et al. [44] showed a reduction of aggressive behaviors and skin lesions in enriched environments. The reasons for the discrepancy of the effects of enrichment on behavior are not clear, but most likely relate to the characteristics of the particular environment with differences in space allowance, complexity of enrichment, feeding and outdoor access. The absence of an effect of enriched housing on total aggressive and total sexual behaviors also explains the absence of an effect on penile injuries and the little effect of skin lesions in IC and EM.

In sum, the results of the present study point to a more favorable effect of ENR over STD housing in all three sex categories. It should, however, be taken into account that this beneficial effect was neither pronounced nor observed in all stages of the animals’ lives. This outcome only partially meets the hypothesis that enriched housing would have beneficial effects in EM on behavior-related welfare aspects including penile injuries. It is quite possible that the additional space and the enrichment were not sufficient to reduce the undesired consequences of sexual and aggressive behaviors on penile injuries. Moreover, enriched environments may even stimulate mountings and aggression by EM as shown by Prunier et al. [45], thereby counteracting the desired effect of enrichment. Obviously, one main factor leading to penis biting and other undesired behaviors in EM is the sex-specific hormonal condition around and after puberty [9], which can be substantially influenced by immunocastration.

Considering the overall discrepancy across studies of the effects of enrichments on aggressive and sexual behavior of boars, it is evident that comparative studies are needed to evaluate the effect of enrichment on the behavior of IC. This is particularly true for the time when IC physiologically and behaviorally switch from entire males to castrates. Certainly, the optimal structure of the physical and social environment differs between boars and IC after the second immunization.

In practice, the second Improvac^®^ vaccination is administered 4–6 weeks prior to slaughter [6,7,15,17,46]. Before the second vaccination, IC can behaviorally and physiologically be considered as boars, which implies that the welfare benefit of IC starts only after V2. An earlier second vaccination would already reduce the number of sexual behaviors at an early stage in the fattening period [19]. Positive effects of immunocastration on animal welfare could be used to its full potential and would come into effect throughout the whole fattening period.

Noteworthy is that the behavior of mixed animals did not substantially differ from non-mixed animals in the present report. This is not surprising because behavioral observations were conducted more than 24 h after the mixing events. The decline in aggressive behavior compared to pre-mixing values is a general observation in male and female pigs and reflects the resettlement of dominance hierarchies [47]. The focus of this study was to observe the accumulating effects of repeated stressor exposure on behavior and welfare, to portray the fattening period with a practical orientation, not the influence of an acute stressor as such. It is, however, well known that during or immediately after mixing intensive aggressive interaction takes place [48,49], which is also confirmed by our own additional observations during the first hour after mixings (Supplementary Material, Table S5). The well-studied increase of agonistic behaviors due to social conflict may be one reason why mixed EM tended to have more penile injuries at slaughter than animals of the other treatment combinations. The unfavorable effect on penile injuries warrants further investigation because the time lag of 3–4 weeks between the second mixing period (P3) and slaughter may have diminished the immediate effect of mixing, especially with respect to the number of fresh wounds. One could expect a larger number of penile injuries if EM would have been slaughtered immediately after the mixings.

The companion study of Kress et al. [16], as well as other studies [50,51], have shown that social mixing of pigs generally has a negative impact on growth performance, presumably because this condition is stressful for the animals and increases lipolytic processes [52]. The present study investigated in more detail the specific relationship between individual behavior and body mass (and some endocrine measures) in all three housing environments. Few significant relationships were found, but they explained the variability of the response data only to a low degree. This leads to the conclusion that a high degree of behavioral and physiological heterogeneity among the individuals exists under all housing conditions assessed in this study. Other studies showed that more severe stressors affect antibody response, as amply reported in rats [53], sheep [54], pigs, cattle [55,56] and mammals in general [57]. Thus, it would be of interest to study the contribution of severe stressors to the “non-responder” phenomenon, which is sometimes reported in otherwise properly vaccinated animals [58,59,60,61].

## 5. Conclusions

Taken together, the results show that throughout the whole fattening period, EM express higher frequencies of undesired fighting and sexual behaviors, which can reduce animal welfare regardless of the degree of enrichment. In any case, the present study reveals that the positive effects of the second (=effective) Improvac^®^ vaccination on problematic behaviors such as sexual behavior, fighting, mounting, and penis biting persist in immunocastrates, even if housing conditions vary. Further studies are needed to assess the modulating effects of housing conditions on immunocastrates analyzing more severe stressors.

## Figures and Tables

**Table 1:** Lesions score assessment carried out for every pig after Schrader et al. [35].

Body Part	Lesion Score	Number of Lesions
Right/left torso	0	<4 line-shaped scratches with a length of ≥5 cm and no extensive injury with a diameter of ≥2.5 cm
	1	4–15 line-shaped scratches with a length of ≥5 cm and no extensive injuries with a diameter of ≥2.5 cm
	2	>15 line-shaped scratches with a length of ≥5 cm or one extensive injury with a diameter of ≥2.5 cm
Right/left ear	0	Only line-shaped scratches on the outside of the ear and no clearly visible bleeding wounds or crusts
	1	Clearly visible, mostly bleeding wounds or crusts on the ear (particularly occurring at the tip, edge or bottom of the ear)
Tail	0	No clearly visible bleeding wounds, crusts or swelling on the tail
	1	Clearly visible bleeding wounds, crusts or swelling on the tail

**Table 2:** Number of penis biting events per animal (mean ± std) in two different behavior observation phases (PreV2: P1+P2; PostV2: P3+P4) displayed for the two relevant sex categories (EM = entire males, IC = immunocastrates).

Sex	PreV2	PostV2
EM	0.16 ± 0.68	0.20 ± 0.47 ^a^
IC	0.23 ± 0.66 ^A^	0.06 ± 0.29 ^bB^

^a,b^ Different small letters indicate differences between sex categories within PostV2 (*p* = 0.039); ^A,B^ Different capital letters denote differences between time points within the sex category IC (*p* = 0.036). Differences were calculated using Wilcoxon signed-rank test.

**Table 3:** Number of penile injuries per animal (Lsmeans ± SEM) for sex categories entire males (EM, n = 48) and immunocastrates (IC, n = 48) and the interaction of sex and housing (ENR = enriched: n = 12; MIX = mixing: n = 24; STD = standard: n = 12 per sex category).

Sex	Number of Penile Injuries (Lsmeans ± SEM)	Interaction Sex × Housing	Number of Penile Injuries (Lsmeans ± SEM)
Entire males (EM)	3.79 ± 2.04 ^a^	EM ENR	3.18 ± 1.94
		EM MIX	6.12 ± 3.38
		EM STD	2.79 ± 1.68
Immunocastrates (IC)	1.50 ± 0.81 ^b^	IC ENR	1.57 ± 0.96
		IC MIX	1.81 ± 1.00
		IC STD	1.18 ± 0.73

^a,b^ Different letters indicate significant differences between sex categories (*p* < 0.001).

## Data Availability

The data presented in this study are available on request from the corresponding author.

## Supplementary Materials

The following materials are available online at https://www.mdpi.com/2076-2615/11/3/618/s1, Table S1: Ethogram of scored behaviors, Figure S1: Highly aggressive behavior, Figure S2: Highly sexual behavior, Table S2: Linear regression analysis for certain behaviors with kg weight, Table S3: Linear regression analysis for certain behaviors with ng/ml cortisol, Table S4: Linear regression analysis for certain behaviors with ng/ml testosterone, Table S5: Mixing data.
