# Supplementary material for: Impact of Housing Condition on Welfare and Behavior of Immunocastrated Fattening Pigs (Sus scrofa domestica)
Source: Animals (Basel). 2021 Feb 26;11(3):618. doi: 10.3390/ani11030618 (PMC7996791; doi:10.3390/ani11030618)
Supplement: Supplementary file 1 [file animals-11-00618-s001.pdf]

animals

Supplementary Material

Impact of Housing Condition on Welfare and Behavior of Immunocastrated Fattening Pigs (*Sus scrofa domestica*)

Linda Steybe [1,*], Kevin Kress [1,2], Sonja Schmucker [1] and Volker Stefanski [1]

[1] Department of Behavioral Physiology of Livestock, Institute of Animal Science, University of Hohenheim, Garbenstraße 17, 70599 Stuttgart, Germany; kress.kevin@uni-hohenheim.de (K.K.); sonja.schmucker@uni-hohenheim.de (S.S.); volker.stefanski@uni-hohenheim.de (V.S.)
[2] German Genetic, Schweinezuchtverband Baden-Wuerttemberg e.V., Im Wolfer 10, 70599 Stuttgart, Germany; kress@german-genetic.de (K.K.)
* Correspondence: linda.steybe@uni-hohenheim.de

Citation: Steybe, L.; Kress, K.; Schmucker, S.; Stefanski, V. Impact of Housing Condition on Welfare and Behavior of Immunocastrated Fattening Pigs (*Sus scrofa domestica*). *Animals* **2021**, *11*, x. https://doi.org/10.3390/xxxxx

Academic Editor: Frank Dunshea and Surinder Singh Chauhan

Received: 1 February 2021
Accepted: 21 February 2021
Published: date

Publisher's Note: MDPI stays neutral with regard to jurisdictional claims in published maps and institutional affiliations.

Copyright: © 2021 by the authors. Submitted for possible open access publication under the terms and conditions of the Creative Commons Attribution (CC BY) license (http://creativecommons.org/licenses/by/4.0/).

Table S1: Ethogram of scored behaviors. A new behavior was scored after 3 seconds of pausing a previous behavior.

**Total Aggressive Behavior** (Jensen, 1980 and Donaldson et al., 2002)	
Reciprocal pressing	The pigs stand parallel or inverse parallel and push hard with the shoulders against each other, throwing their heads against the neck, head or flanks of the other pig.
Reciprocal pressing-cum-bite	As above, but with bites directed towards the head, ears and flanks of the other pig.
Head knock	A rapid thrust upwards or sideways with the head or snout against any part of the body of the other pig. The performing pig's mouth is shut.
Head knock-cum-bite	As above, but with bites. The performing pig's mouth is open.
Biting (attempt)	The pig's mouth is being opened and snapped shut against an opponent.
Penis biting	Biting towards the extruded penis of another pig.
Levering	The pig puts its snout under the body of another pig (in all observed cases from behind) and raises its snout to topple the other pig.
Chasing	Following a fleeing animal at high speed.
Total defensive behavior (modified after Jensen, 1980 and Camerlink et al., 2015)	
Retreat	The pig moves away from another pig at its usual walking speed directly after a social interaction.
Fleeing	The pig moves away from another pig rapidly with its head held high directly after a social interaction. This is often accompanied by a shrill scream.
Mounting escape (attempt)	Occurs in response to mounting. The pig tries to or moves away from the mounting pig rapidly. The activity is often accompanied by a shrill scream.
Social nosing behavior (modified after Jensen, 1980 and Camerlink et al., 2015)	
Nosing	The nose of the pig approaches any body part except the genital region of another pig up to at least 5 cm distance. No agonistic behaviors follow for at least 3 seconds.
Total sexual behavior (modified after Booth and Baldwin, 1980)	
Mounting attempt	The pig lifts the front part of its torso to put it on top of the torso of another pig (usually from behind), but is not successful.
Mounting	The pig lifts the front part of its torso and puts it on top of the torso of another pig (usually from behind).
Mounting with pelvic thrusts	While maintaining the mounting position, the pig moves its pelvis for- and backward.
Mounting with extruded penis	While maintaining the mounting position, the pig extrudes its penis.
Mounting with pelvic thrusts and extruded penis	While maintaining the mounting position, the pig moves its pelvis for- and backward and extrudes its penis.
Anal-genital-nosing (modified after Jensen, 1980)	The nose of the pig approaches the genital region of another pig up to at least 5 cm.
Play behavior (Donaldson et al., 2002)	
Scamper	A sequence of at least two forward hops in rapid succession, usually accompanied by ear flapping.
Other play	Pivot (a jump on the spot in which the pig rapidly turns its body at least 90° in the horizontal plane), head toss (exaggerated lateral displacements of the head and neck, involving at least one full movement to each side), flop (the pig rapidly falls down from an upright position into sternal or lateral recumbency on its own initiative without contact with another pig).
Abnormal behavior (modified after Jensen et al., 2010)	
Chewing pen mate	The pig chews at a body part of a (mostly lying) pen mate, except ears and tail.
Chewing tail	The pig chews with the tail of another pig in its mouth.
Chewing ear	The pig chews with the ear of another pig in its mouth.
Body-Nosing (modified after Fraser, 1978)	The rhythmic up-and-down movement of one pig rubbing the body, especially the belly, of another with its snout.

Booth, W.D., Baldwin, B.A. (1980). Lack of effect on sexual behaviour or development of testicular function after removal of olfactory bulbs in prepubertal boars. Journal of Reproduction and Fertility 58: 173-182.

Camerlink, I. et al. (2015) "Aggressiveness as a component of fighting ability in pigs using a game-theoretical framework", Animal Behaviour, 108, pp. 183–191. doi: 10.1016/j.anbehav.2015.07.032.

Donaldson, T.M.; Newberry, R.C; Spinka, M.; Cloutier, S. (2002). Effects of early play experience on play behaviour of piglets after

weaning. Applied Animal Behaviour Science 79, 221-231.

Ekkel, E. D..; Spoolder, H.A.M.; Hulsegge, I.; Hopster, H. (2003). Lying characteristics as determinants for space requirements in pigs. Applied Animal Behaviour Science 80, 19-30.

Fraser, D. (1978). Observations on the behavioural development of suckling and early-weaned piglets during the first six weeks after birth. Anim. Behav. 26, 22-30.

Jensen, P. (1980). An ethogram of social interaction patterns in group-housed sows. Applied Animal Ethology 6, 341-350.

Jensen, M.B.; Studnitz, M., Pedersen, L.J (2010). The effect of type of rooting material and space allowance on exploration and abnormal behaviour in growing pigs. Applied Animal Behaviour Science 123: 87-92.

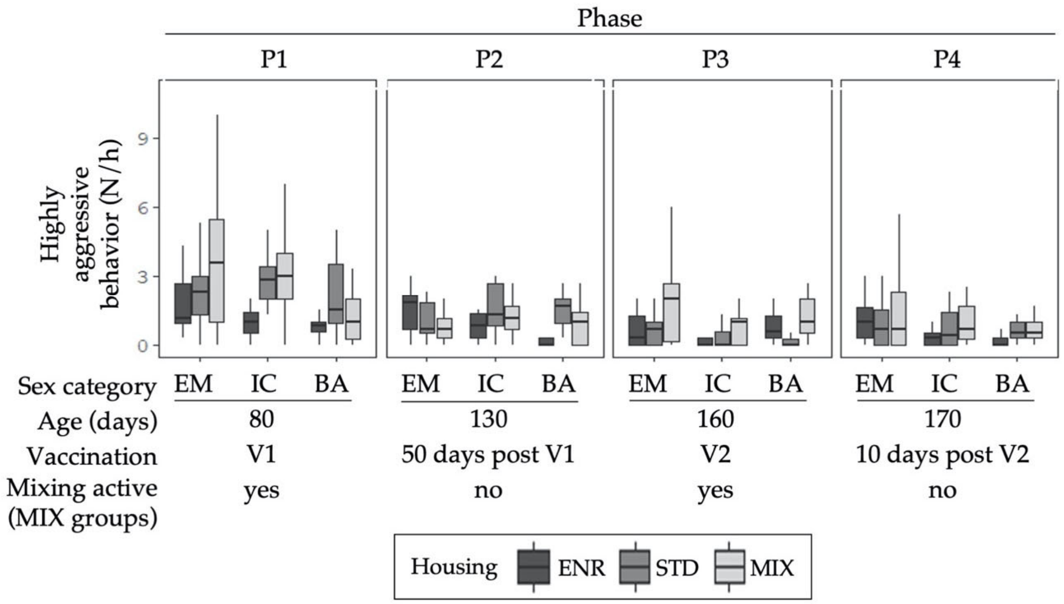

Figure S1. Highly aggressive behavior. Mean number of occurrences (N) of highly aggressive behavior per hour (h) for each animal are given as boxplots for the sex groups in the different environments. Columns represent the data of animals at four different ages (P1–P4). V1 represents phases before, and V2 after the second immunization of IC. The MIX groups were mixed in P1 and P3. Note: no mixing was done in STD and ENR at any time. Sex groups were: EM = entire males, IC = immunocastrates, BA = barrows; Housing environments were: ENR = enriched, STD = standard, MIX = mixing. No significant differences were found.

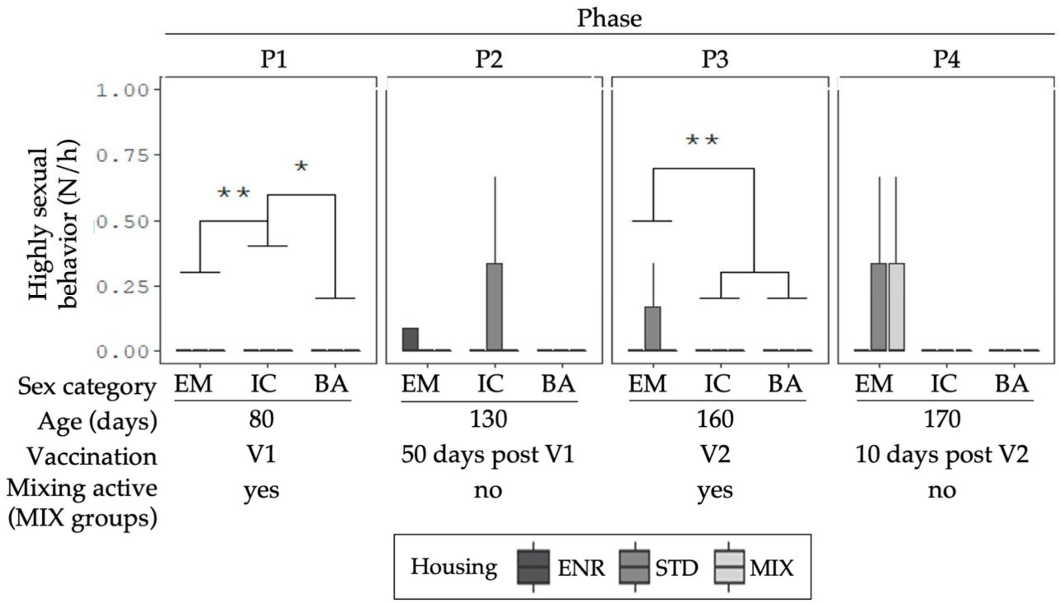

Figure S2: Highly sexual behavior. Mean number of occurrences (N) of highly sexual behavior per hour (h) for each animal are given as boxplots for the sex groups in the different environments. Columns represent the data of animals at four different ages (P1–P4). V1 represents phases before, and V2 after the second immunization of IC. The MIX groups were mixed in P1 and P3. Note: no mixing was done in STD and ENR at any time. Sex groups were: EM = entire males, IC = immunocastrates, BA = barrows; Housing environments were: ENR = enriched, STD = standard, MIX = mixing. Significant differences between sex groups are indicated by asterisks (*** $p < .001$, ** $p < .01$, * $p < .05$).

Table S2. Linear regression analysis for certain behaviors with kg weight. Linear regression equation information ($y_i = \beta_0 + \beta_1 X_i + \varepsilon_i$) with frequencies of behaviors in the last observation phase at about 170 days of age as dependent variable and body weight (kg) at the age of about 180 days (6 months) as independent variable displayed for all 9 treatment combinations (sex: EM = boars, IC = immunocastrates, BA = barrows; housing: ENR = enriched, STD = standard, MIX = mixing). Equations are shown in tabular display.

		**Regression equation information for body weight (kg) as dependent variable (X_i)**								
		**EM**			**IC**			**BA**		
**y_i**		**ENR**	**STD**	**MIX**	**ENR**	**STD**	**MIX**	**ENR**	**STD**	**MIX**
Total aggressive	β_0	2.01	1.40	1.82	1.22	1.46	1.66	1.10	1.31	1.33
$F_{(1,127)} = 1.50$, $p = .223$,<br>$R^2 = .0326$	β_1									
Fighting	β_0	1.70	1.13	1.17	–0.44	–0.45	–0.37	0.72	0.76	0.68
$F_{(3,129)} = 2.44$, $p = .067$,<br>$R^2 = .3132$	β_1		–0.00700			0.00407			–0.00441	
Total sexual	β_0	–0.06	–0.03	0.13	–0.15	–0.02	0.02	–0.14	–0.15	–0.13
$F_{(1,84.9)} = 0.35$, $p = .554$,<br>$R^2 = .0719$	β_1									
Mounting	β_0	0.72	0.65	0.79	0.37	0.46	0.48	0.37	0.38	0.38
$F_{(1,132)} = 1.72$, $p = .192$,<br>$R^2 = .2677$	β_1									
Abnormal	β_0	1.02	1.26	1.06	0.81	0.98	1.09	0.83	1.10	1.01
$F_{(1,123)} = 0.37$, $p = .546$,<br>$R^2 = .0337$	β_1									
Total defensive	β_0	–0.06	–0.03	0.13	–0.15	–0.02	0.02	–0.14	–0.15	–0.13
$F_{(1,84.9)} = 0.35$, $p = .554$,<br>$R^2 = .0719$	β_1									
Mounting escape	β_0	0.28	–2.06	–0.92	0.004	0.04	–0.12	–0.03	–0.12	–0.04
$F_{(9.118)} = 2.47$, $p = .013$,<br>$R^2 = .3155$	β_1	-0.00172	0.01763	-0.00547	-0.00002	-0.00022	-0.00124	0.000280	0.00111	0.000361

Table S3: Linear regression analysis for certain behaviors with ng/ml cortisol. Linear regression equation information ($y_i = \beta_0 + \beta_1 X_i + \varepsilon_i$) with frequencies of behaviors in the last observation phase at about 170 days of age as dependent variable and cortisol concentration (ng/ml) in plasma at blood collection B3 as independent variable displayed for all 9 treatment combinations (sex: EM = boars, IC = immunocastrates, BA = barrows; housing: ENR = enriched, STD = standard, MIX = mixing). Equations are shown in tabular display.

		Regression Equation Information for Cortisol Concentration (ng/ml) as Dependent Variable (X_i)								
y_i		EM			IC			BA		
		ENR	STD	MIX	ENR	STD	MIX	ENR	STD	MIX
Total aggressive	β_0	1.53	0.93	1.39	0.38	0.68	0.94	0.76	1.09	1.01
$F_{(3,116)} = 2.89$, $p = 0.038$, $R^2 = .0231$	β_1		–0.00420			0.01080			–0.01294	
Fighting (C*S)	β_0	0.92	0.46	0.51	-0.05	–0.02	0.06	0.30	0.40	0.27
$F_{(3,123)} = 5.20$, $p = 0.002$, $R^2 = .2908$	β_1		–0.00825			0.00612			–0.00824	
Total sexual	β_0	0.12	0.16	0.30	0.04	0.16	0.18	0.03	0.04	0.04
$F_{(1,131)} = 0.72$, $p = 0.399$, $R^2 = .0881$	β_1					–0.00136				
Mounting	β_0	0.30	0.22	0.38	–0.08	0.02	0.07	–0.05	–0.07	–0.03
$F_{(1,126)} = 2.75$, $p = 0.100$, $R^2 = 0.2369$	β_1					0.00324				
Abnormal	β_0	0.67	0.89	0.70	0.43	0.61	0.74	0.47	0.72	0.67
$F_{(1,129)} = 1.01$, $p = 0.316$, $R^2 = 0.0190$	β_1					0.00357				
Total defensive	β_0	0.12	0.16	0.30	0.04	0.16	0.18	0.03	0.04	0.04
$F_{(1,131)} = 0.72$, $p = 0.399$, $R^2 = 0.0988$	β_1					–0.00136				
Mounting escape	β_0	0.08	0.19	0.30	0.04	0.04	0.08	0.04	0.07	0.03
$F_{(1,131)} = 1.37$, $p = 0.244$, $R^2 = 0.2067$	β_1					–0.00152				

Table S4: Linear regression analysis for certain behaviors with ng/ml testosterone. Linear regression equation information ($y_i = \beta_0 + \beta_1 X_i + \varepsilon_i$) with frequencies of behaviors in the last observation phase at about 170 days of age as dependent variable and testosterone concentration (ng/ml) in plasma at blood collection B3 as independent variable displayed for all 9 treatment combinations (sex: EM = boars, IC = immunocastrates, BA = barrows; housing: ENR = enriched, STD = standard, MIX = mixing). Equations are shown in tabular display.

		**Regression Equation Information for Testosterone Concentration (ng/ml) as Dependent Variable (X_i)**								
**y_i**		**EM**			**IC**			**BA**		
		**ENR**	**STD**	**MIX**	**ENR**	**STD**	**MIX**	**ENR**	**STD**	**MIX**
Total aggressive	β_0	1.41	0.81	1.25	0.64	0.87	1.11	0.52	0.71	0.76
$F_{(1,124)} = 0.22$, $p = 0.642$, $R^2 = -0.0037$	β_1	0.00709								
Fighting	β_0	0.77	0.25	0.32	0.10	0.10	0.16	0.14	0.17	0.12
$F_{(7,121)} = 0.9$, $p = 0.506$, $R^2 = 0.2167$	β_1	0.00593	0.06697	–0.01532	–0.01628	–0.2810	0.5303	0	0	0.6825
Total sexual	β_0	0.15	–0.02	0.25	–0.10	–0.01	0.01	0.04	0.03	0.04
$F_{(5,123)} = 2.28$, $p = 0.051$, $R^2 = 0.1352$	β_1	–0.1315	0.9399	0.00506	–0.05287	1.0186	0.08370	–0.1527	0.9188	–0.01613
Mounting	β_0	0.37	0.30	0.47	0.003	0.10	0.14	0.01	0.02	0.03
$F_{(1,128)} = 0.21$, $p = .645$, $R^2 = 0.2208$	β_1	–0.00400								
Abnormal	β_0	0.72	0.97	0.79	0.52	0.67	0.80	0.54	0.81	0.73
$F_{(7,121)} = 1.26$, $p = 0.278$, $R^2 = 0.200$	β_1	–0.04127	0.06830	–0.00182	5.4750	–0.167	0.9276	0	0	0.2651
Total defensive	β_0	0.15	-0.02	0.25	–0.10	–0.01	0.01	0.04	0.03	0.04
$F_{(5,123)} = 2.28$, $p = 0.051$, $R^2 = 0.1352$	β_1	–0.1315	0.9399	0.005063	–0.05287	1.0186	0.08370	–0.1527	0.9188	–0.01613
Mounting escape	β_0	0.03	0.14	0.23	0.002	0.004	0.05	0.01	0.03	0.004
$F_{(1,129)} = 1.73$, $p = 0.191$, $R^2 = 0.2178$	β_1	0.00747								

Table S5: Mixing data. Mean number of received total aggressive behavior per animal in MIX groups in the first hour after mixing, with numbers given separately for resident and intruder animals, as well as on a regular observation day at least 24 h after the mixing event, giving the mean of all animals in the group. Both observations took place in the first observation phase (P1: Pigs are about 80 days old, mixing carried out in the MIX groups). Mean ± standard deviation is given.

		**Numbers of Total Aggressive Behavior Received per Animal (Mean ± SD)**	
**Sex Category**	**Status of the Animal**	**First Hour after Mixing**	**Regular Observation Day**
EM	Resident	6.88 ± 13.8	4.79 ± 3.75
	Intruder	14.6 ± 14.1	
$IC_{(V1)}$	Resident	9.25 ± 8.36	3.88 ± 2.71
	Intruder	18.1 ± 12.8	
BA	Resident	5.62 ± 4.76	2.40 ± 2.55
	Intruder	14.4 ± 11.8	
